# Exploring patients’ experiences with telehealth in obstetrics care during the COVID-19 pandemic: A qualitative study

**DOI:** 10.1371/journal.pone.0292799

**Published:** 2023-12-20

**Authors:** Mohammad Alkawaldeh, Asma Alkhawaldeh, Tracy Yeboah

**Affiliations:** 1 College of Nursing, QU Health, Qatar University, Doha, Qatar; 2 Research Specialist, Jordanian Royal Medical Services, Amman, Jordan; 3 Department of OBGYN, Research Coordinator, University of Massachusetts Medical School, Worcester, MA, United States of America; Sefako Makgatho Health Sciences University, SOUTH AFRICA

## Abstract

**Aim:**

The aim of this study was to evaluate patients’ experiences with telehealth provision of obstetrics and gynecology care during the COVID-19 pandemic qualitatively.

**Design and setting:**

In this study, a qualitative research design, namely descriptive phenomenology, was employed. Participants were recruited from the OB department at UMass Memorial Medical center in Worcester, MA, between 6/2020 and 7/2020.

**Methods:**

Between June 2020 and July 2020, in-depth interviews were conducted with 18 women receiving care at the Obstetrics and Maternal and Fetal Medicine clinics. Data were analyzed using qualitative thematic analysis, as outlined by Braun and Clarke.

**Results:**

Telehealth is a feasible and safe health-care tool that is available during these unprecedented times. This study provided qualitative evidence based on patients’ perspectives and experiences. Participants’ meanings in relation to their experiences of using telehealth services emerged from the data in four themes: the overall experience of using modern telehealth platforms, telehealth and its perceived benefits, telehealth and its perceived challenges, and telehealth and its potential future use.

**Conclusion:**

While this study highlights areas in telehealth implementation that require improvement, the overall positive experiences and consistent perceived benefits of most participants suggests that telehealth can be an important tool in healthcare delivery for appropriate patients and situations moving forward in a post-pandemic world.

**Impact:**

During the global pandemic, telehealth has been recognized to have the potential to play a critical role in healthcare delivery. Establishing qualitative evidence-based practices in the emerging field of telehealth for OB services is pivotal to mitigate potential safety, feasibility, and cost issues that could be associated with the rapid adoption of telehealth. Yet, this qualitative study However highlighted several challenges that are necessary to be addressed in order for telehealth to meet maximum effectiveness and functionality in the future.

## Introduction

Access to obstetric (OB) health-care services can affect the quality of life of women and their families at an individual level and society at a national level. In the United States, during the COVID-19 pandemic, OB patients faced unique challenges, including the decrease of prenatal visits, high frequency of maternal mental health problems (e.g., anxiety and depression), domestic violence, women being more vulnerable to lose their income due to the pandemic than men, and women struggling with childcare and receiving less social support [1]. These challenges, if not promptly addressed, create poor health outcomes, particularly for high-risk OB patients and underserved female populations. Obstetric health-care practices were then advised by the American College of Obstetrics and Gynecology (ACOG) to include telehealth models in their services, to attain the continuity of providing women with the care they need during this challenging time.

Telehealth technology and its use are not new concepts in the field of medicine in general and OB in particular. Yet, the use of telehealth in OB practices was minimal in comparison to other practices in the United States [2]. During the global pandemic, telehealth has been recognized to have the potential to play a critical role in health-care [3–5]. Telehealth is described as a collection of means or methods for enhancing the health care, public health, and health education delivery and support, by allowing physicians and patients to interact virtually, using various telecommunications technologies, including videoconferencing, store-and-forward imaging, the internet, streaming media, and wireless and terrestrial communication [6,7]. More specifically, telehealth has been used for various topics, including diagnosis and management, education and training, consultation, surveillance, and to provide an umbrella term for the various novel emerging technologies used to improve the delivery of health-care services [7–9].

### Background

In the field of OB, telehealth services have been expanded to every aspect of women’s health. Examples include remote observation of ultrasound recordings by a maternal-fetal medicine [10], remote blood pressure monitoring [11], childbirth education [12], fertility tracking with patient-generated data [13], medication management and consultations [14], bladder diary tracking with smartphone apps [7], and virtual patient consultation with specialty services [15]. Additionally, telehealth has played a critical role during the pandemic, by reducing disease exposure to patients and providers, expanding access to care [16], facilitating social distancing measures [5,17,18], and minimizing the impact of patient demands on facilities [16,19]. Furthermore, through the use of telehealth services, health-care organizations have preserved valuable personal protective equipment supplies (PPE) for direct contact encounters while allowing providers to administer care via telehealth to patients in need [18,20].

Additional studies to understand the key tenets of evidence on feasibility, safety, cost, perceived benefits, and barriers in the field of OB more comprehensively are required, particularly the qualitative efforts to address patients’ perceptions and experiences of the use of telehealth. Establishing qualitative evidence-based practices in the emerging field of telehealth for OB services is pivotal to mitigate potential safety, feasibility, and cost issues that could be associated with the rapid adoption of telehealth.

## The study

### Aim

The aim of this study was to evaluate patients’ experiences with telehealth provision of obstetrics and gynecology care during the COVID-19 pandemic qualitatively. We particularly aimed at exploring patients’ experiences and their perceptions of telehealth’s perceived benefits, challenges, and potential use in the post-pandemic world.

### Design

In this study, a qualitative research design, namely descriptive phenomenology, was employed. Furthermore, qualitative thematic analysis, as outlined by Braun and Clarke [21], was employed to study OB patients’ experiences and perceptions of using telehealth services during the COVID-19 pandemic.

### Sample and settings

A purposive sampling method was employed in this study. Patient participants were recruited from the UMass Memorial Medical Health Center in Central Massachusetts. The inclusion criteria were (a) OB patients who attended at least one telehealth visit during the COVID-19 pandemic, (b) able to read and speak English, and (c) willing to participate in a 60-minute virtual individual interview. Exclusion criteria were (a) inability to provide verbal consent, (b) inability to speak English, and (c) patients who never attended a telehealth visit.

A list of potential patient participants was collected from the OB daily scheduled outpatient list. Eligible patients who met the inclusion criteria were contacted by a research assistant, to discuss participation in the study. The interviews were completed remotely, using a secure and health insurance portability and accountability (HIPPA) compliant platform, namely Zoom. In total, 38 OB patients were screened for eligibility, whereby nine patients did not meet the inclusion criteria, as they never attended a telehealth visit. Eleven patients either did not respond to the invitation or were unavailable during the interviews schedule. Eighteen OB patients agreed to participate in the study and completed the virtual interviews.

### Data collection

Data was collected using a semi-structured in-depth interview technique. From June to July 2020, all patient interviews were conducted by the principal investigator (PI), MA. The PI exhibits robust qualitative research expertise and had conducted various qualitative research studies. To assure the acquisition of rich and trustworthy data, the PI built rapport with participants prior to interviews and used various techniques during the interviews including repetition, follow-up inquiries, and summarizing while avoiding the researcher’s subjectivity biases. Prior to the actual data collection, the principal investigator conducted two short pilot interviews. The purpose of the pilot interviews was to test interview questions and identify any challenges that might be encountered during the actual data collection. For the actual data collection, the 18 interviews were conducted remotely, using the Zoom teleconferencing platform, each lasting 30 minutes on average. All interviews were audio-recorded on Zoom. The principal investigator was responsible for conducting the interviews. Prior to conducting the interviews, the study procedure was explained and verbal consent was obtained. The semi-structured interview questions were developed by the principal investigator and reviewed by the co-investigator (TMS), according to the study methodology and research question, with open-ended questions. The interview questions focused on six factors, including overall experience and satisfaction, perceived benefits, perceived challenges, technical aspects, expectations, and patient-provider connections. Reflective journals were also used by the researcher in this qualitative study as a methodological tool to promote reflexivity and self-awareness throughout the research process. After the completion of each interview, the principal investigator completed a short form including reflective notes describing initial findings, interpretation, and nonverbal cues. Participants’ interviews continued until interview information reached saturation. Additionally, a short electronic survey on the demographic and general characteristics (including age, race, ethnicity, previous use of telehealth, use of technology forms, gravidity, and parity) was completed by each participant via a REDCap link. The data from the notes and individual interviews were subsequently transcribed and made available for analysis.

### Ethical considerations

The study was approved by the University of Massachusetts, Worcester Institutional Review Board. To maintain participants’ privacy and confidentiality pseudonyms were assigned to each individual in this study. Prior to conducting individual interviews, participants received a comprehensive overview of the study’s objectives, significance, and importance of confidentiality and privacy. Each participant’s informed consent was then obtained, ensuring their voluntary involvement in the study.

### Data analysis

Patients’ demographic characteristics were summarized and detailed using descriptive statistics, including frequencies and percentages. All interviews were transcribed verbatim in their entirety. Interview transcriptions, notes, and reflective journals were uploaded into Dedoose version 8.9 qualitative software to support data organization, the development of codes, and the construction of categories to aid in the coding process. The PI used an inductive methodology allowing the derived themes to emerge spontaneously from the data. The data analysis was guided by the six phases thematic analysis approach as outlined by Braun and Clarke [21] and reviewed by two research members (MA and TMS) (Table 1).

**Table 1:** Thematic analysis.

Phase	Analysis description
Phase 1: Familiarization of the data	Read the data line by line multiple times, and any initial ideas and interesting features of the data will be noted.
Phase 2: Generating initial codes	Assign codes (referred to as parent and child codes in Dedoose) to interesting features of the data. The coding will be completed systematically across the entire dataset. Gathering the data relevant to each potential code.
Phase 3: Searching for themes	Review the initial codes and cluster them under larger themes. Gathering the data relevant to each potential theme.
Phase 4: Reviewing themes	Check themes application in relation to the coded extracts and the entire data set (Reduce the number of codes, eliminate duplication). Generate a thematic map of the analysis.
Phase 5: Defining and naming themes	Ongoing analysis to refine the specifics of each theme and the overall narrative by the analysis. Generate clear definitions and names for each theme.
Phase 6: Producing the report	Select vivid, compelling extract examples. Final analysis and produce a scholarly report.

### Rigor

To enhance the trustworthiness of the qualitative data during the collection and analysis process, four criteria were suggested by Lincoln and Guba [22] and were used in this study, namely transferability, credibility, confirmability, and dependability. Transferability in this study was enhanced by the in-depth nature of 18 interviews. Credibility was assured by using reflective journals, notes, and verbatim transcripts. Confirmability was achieved by having a trained research assistant review all transcriptions by listening to the audio and reading the transcript prior to data coding. Five participants were asked to review their own transcripts, which resulted in no change. Additionally, research members reviewed the emerged themes multiple times until the entire team was satisfied with the final findings. Dependability was achieved by using qualitative data analysis software (Dedoose), to systematically analyze the data, track the frequency of the codes, and create a visual thematic map.

## Findings

### Participant characteristics

As described in Table 2, most of the participants were women under the age of 42. Thirty-nine percent of the participants were receiving prenatal care, (n = 7), and 61% (n = 11) were receiving postnatal care. The majority of the participants were White, non-Hispanic or Latino (39%, n = 7), and White, Hispanic, or Latino (28%, n = 5). All participants reported using at least one form of technology (e.g., smartphone, computer, or tablet) and had experience with telehealth visits, either using only audio or video.

**Table 2:** Participant demographic characteristics (n = 18).

	n (%)
Age (yrs) 25–29 30–35 36–42	3 (17%) 7 (39%) 8 (44%)
Ethnicity	
Hispanic or Latino	5 (28%)
Not Hispanic or Latino	13 (72%)
Race	
Black/African American	2 (11%)
White	12 (67)
Asian	3 (17%)
Other/not specified	1 (5%)
Marital status Single Divorced Separated Married	6 (34%) 1 (5%) 1 (5%) 10 (56%)
Gravidity 0 1 2 >2	3 (17%) 7 (39%) 7 (39%) 1 (5%)
Parity 0 1 2 >2	5 (28%) 7 (39%) 5 (28%) 1 (5%)

### Themes and sub-themes

During the interview sessions, study participants discussed their experiences of utilizing telehealth care services during the COVID-19 pandemic, including overall experiences, perceived benefits, challenges, and future potentials of telehealth in OB practices. At the end of the interview sessions, positive comments such as “wonderful topic,” “excellent health-care services,” and “positive conversation” indicated that the participants felt they were respected, valued, and cared for during this challenging time. Participants’ meanings in relation to their experiences of using telehealth services emerged from the data in four themes (Table 3): (1) the overall experience of using modern telehealth platforms, (2) telehealth and its perceived benefits, (3) telehealth and its perceived challenges, and (4) telehealth and its potential future use.

**Table 3:** Major themes and sub-themes.

Major Themes	Sub-themes
1. The overall experience of using modern telehealth platforms	1.1. Ease of Use 1.2 Positive Experiences 1.3 Patients Experiences Based on Age and Digital Literacy
2. Telehealth and its perceived benefits	2.1 Access to Health-Care Services 2.2 Convenience 2.3 Risk Reduction
3. Telehealth and its perceived challenges	
4. Telehealth and its potential future use	4.1 Follow-Ups 4. 2 Counseling

### Theme one: 1. Overall experience of using modern telehealth platforms

Participants’ overall experiences and perceptions of using modern telehealth platforms are described in the following sections in detail, based on the ease of use, positive experience, age, and digital literacy.

#### Subtheme: 1.1 Ease of use

During this pandemic, technological applications such as telehealth platforms emerged as effective and sustainable solutions for patients to meet their health-care needs. In this study, most participants acknowledged the ease and the simplicity of connecting with their providers using the telehealth platform. For example, Suzanne, a 41-year-old patient, expressed her excitement for the ease of use of telehealth, stating that

*“I can use whatever the type of smart device is, computer, iPad, iPhone, to connect with my provider using the telehealth link. It was very easy.”*

The current pandemic has uncovered the potential impact of digital life and made it clear how virtual platforms may become more incorporated into people’s daily lives. Individuals worldwide use various forms of technologies, and an increasing number of individuals are becoming familiar with various teleconferencing tools. In this study, various forms of technological applications were reported to be used during the pandemic for social connections purposes, such as Zoom, Google Duo, Avidia, Facetime, Skype, WhatsApp, WebEx, Viber, and Google Hangouts. Shannon, a 39-year-old patient, stated,

“*We often use WebEx for meetings*, *and then during the pandemic*, *friends were using zoom calls*, *and we were also using another app called Google Hangouts*. *Also*, *I have used different apps for chatting with friends*.*”*

#### Subthemes: 1.2 Positive Experiences

The vast majority of the study participants positively described their experience with telehealth as “very helpful,” “quick,” “exciting,” great service,” and “super easy.” Deanna, a 40-year-old prenatal patient, stated that

“*It was totally enlightening to have this telehealth visit*. *Who knew telehealth appointments could be that easy*?*”*

Additionally, participants expressed feeling valued, listened to, and cared for during their telehealth visits. Marta, a 28-year-old patient stated,

*I went through my questions with her [referring to provider]*, *and she made me feel like she had time to answer all my questions as if I was in person*. *I don’t think I would have asked or said anything different if I was physically in person with her versus doing the video visit*.

Similarly, Kimberly, a 30-year-old patient, reflected positively on the overall experience, stating, “My visit went pretty well, she listened to me [referring to provider] explaining what was going on. She was very knowledgeable about me, so I felt pretty good after that.”

#### Subthemes: 1.3 Patients experiences based on age and digital literacy

It seems the older patients often lack basic training in utilizing technology, so they may find the telehealth platforms to be frustrating. When discussing her feedback regarding her experience with telehealth, Leona stated:

*I*’*m 42 years old*, *and I relied on my husband to do most of that for me*! *I’m not a computer girl; you are lucky I knew how to get on my telephone*! *I can use the internet*, *but it’s very frustrating*.

In contrast, telehealth tends to be easier for young patients to comprehend since they are regularly immersed with modern technology. Meagan, a 26-year-old patient, said,

*“I had no issues at all*. *I was able to figure it out on my own. Pretty easy.”*

### Theme two: 2. Telehealth and its perceived benefits

During COVID-19, telehealth represents a unique approach to provide necessary health-care services. The perceived benefits of telehealth visits were expressed with regards to health-care services, convenience, and risk reduction.

#### Subtheme: 2.1 Access to health-care services

In an effort to reduce unnecessary patient exposure to the coronavirus, ACOG recommended that non-urgent concerns and regular checkups be canceled, postponed, or converted to telehealth appointments [23]. Thereby, the majority of the patients in this study were satisfied with the telehealth services, as they could access a specialized OB provider and discuss their health concerns from the safety of their homes. Ana, a 30-year old mother stated,

*You know*, *I liked the fact that I was able to have a discussion*, *get more information*, *or touch base with my specialist from the comfort of my home*. *I found it to be nice and convenient*, *especially during this difficult time*. *To be honest*, *I was worried*, *I won’t be able to see my provider for a long time*.

#### Subthemes: 2.2 Convenience

Traditionally, patients have to make several arrangements in order to complete an in-person visit. These individuals may need to take time off from work or school, seek alternative childcare arrangements, and coordinate transportation to/from the doctor’s office. Additionally, they will likely have to undergo check-in processes and long waiting times. Most study participants concluded that telehealth was significantly more convenient than an in-person visit for the following reasons. First, telehealth provides patients with the ability to attend doctor’s visits from home or any chosen location. Thus, it eliminates the hassle of commuting, parking, in-person wait times, and in-person physical contact. Participants expressed how convenient it is to complete a telehealth visit from any convenient location without having to physically commute to an in-person visit. Ana, a 30-year-old patient, stated,

*“It was nice to be able to do this visit from the comfort of my home or from anywhere I want*.”

Second, telehealth saves time. Using telehealth platforms, patients are able to avoid long check-in processes and no longer needed to worry about sitting in a waiting room. Telehealth gives patients the luxury of simply picking up the phone or clicking on a telehealth link and calling their provider. Rosiane, a 41-year-old patient said,

*If you have an appointment at 3 o’clock*, *you get there at 2*:*30*, *and you have to wait for 30 minutes*. *But for the telehealth visit*, *they call you at exactly the time; you don’t even get worried because you know that they will call you/connect with you*.

Similarly, Brittany, a 41-year-old patient, stated,

“*It’s just really convenient to pick up your phone or hop on the computer at the appointed time*, *and the provider was there*. *So*, *it was very timely*.*”*

Third, telehealth allows for one-on-one discussions with providers. By allowing patients to ask all of their questions in a focused and individualized setting, participants found that telehealth has the power to foster more efficient conversations between themselves and the provider. Kristen, a 29-year-old patient, noted,

“*I felt like the visit was much more focused*. *It was completed within 20 minutes*.*”* Similarly, Darelis, a 28-year-old patient, stated,

*I certainly walked away from this visit feeling really good about things*. *So, she [referring to provider] definitely spent plenty of time with me, focusing on my concerns, and made sure all my questions were answered, which I thought was great*.

Fourth, telehealth has the potential to reduce the financial burdens on patients. Requesting time off, fueling a vehicle, and arranging for childcare can lead to financial stress. Telehealth visits can be convenient to patients and fit into a busy schedule. Deanna, a 40-year-old patient, said,

“*It’s very convenient because it’s from the comfort of your home or wherever you are*. *You don’t have to move*, *you do not have to drive a car*, *you don’t have to burn any gas*, *so it’s very convenient*.*”*

#### Subthemes: 2.3 Risk reduction

During the pandemic, telehealth has become a practical health-care tool, ideal for providing treatment without unnecessary exposure. Markedly, the majority of patients reported that using telehealth during this challenging time was very important in reducing their risk of contracting the virus. Patients were grateful for the alternative to in-person visits, as it meant they would be less likely to spread the virus to loved ones after attending an appointment. Allison, a 33-year-old mother, expressed her fear of going to an in-person visit, preferring to complete the visit virtually; she stated,

*In regard to the COVID-19*, *I preferred doing the telephone conversation*. *Again*, *you know*, *they tell us that the hospitals are safe*, *and their colleagues clean this place*, *but it’s also downtown*, *and you don’t know how many people you’re interacting with*, *so I was very excited that telehealth was an option*.

Additionally, patients expressed their concern that another wave of the virus could impact the region. Julie, a 39-year-old patient, said,

*Yeah*, *you know if you run into another quarantine or something*, *obviously yes*, *I’d rather do that [telehealth visit]; my daughter is a high risk*, *so I don’t like having to go out if I don’t have to go*, *I mean that’s the plus to it*, *you know*, *obviously limiting exposure*.

During the pandemic, an in-person visit could easily increase one’s risk of exposure to the virus via touching contaminated surfaces and/or interacting with other patients, office staff, and providers. Patients expressed enormous gratitude for the fact that telehealth allowed them to virtually communicate with providers without physical interaction. Amanda, 36-year-old mother, expressed her appreciation of the telehealth services, stating,

*“If I need to go to the doctor*, *my kids can be exposed to the virus or whatever sickness in the hospital*. *So*, *I think in these circumstances*, *it is better to have a video call appointment*.”

Similarly, Katrina, a 39-year-old new mother, stated,

I did like that I didn’t have to go to the office because, at that point, I think everyone was super scared about coronavirus. You can get it from just touching surfaces, and all this information was coming out about COVID; it was kind of freaking me out especially being pregnant.

### Theme three: 3. Telehealth and its perceived challenges

While telehealth has proven to be an extremely advantageous health-care tool during the pandemic, improving the provision of health services, some patients believe there are some challenges, particularly in obstetric practices. First, participants expressed that lack of digital infrastructure (e.g., wireless blood pressure monitors, glucometers, and activity trackers) is one of the common challenges with telehealth, particularly when remote monitoring is needed. For example, Darelis, a 28-year-old patient, stated,

*My physician wanted me to have a scale and a blood pressure cuff*. *Luckily*, *we have both*, *but I was kind of shocked that she expected us to have those*. *The average person likely does not own standard medical equipment*, *such as a heart rate monitor or a blood pressure cuff*, *nor would they have the training to make adequate blood pressure readings in most scenarios*.

Second, high-risk, chronically ill, pregnant, or postpartum patients often require physical exams and regular checkups that are not currently supported by telehealth functionality. For example, Ana, a 30-year-old patient, stated,

*It was nice to be in the comfort of my home*. *There wasn’t anything medically overwhelming*, *so it was okay*. *But*, *if I had a condition that [required me] to be seen*, *then I would want to see the doctor in an in-person-visi*t.

Third, participants expressed frustration with technical issues. While attending a telehealth appointment, some patients experienced extended buffering times and network disruption issues. Julie, a 39-year old mother, recalled,

*… “the video cut out… [the screen froze] and it never unfroze*.”

Fourth, patients who lack basic training in utilizing technology may find the telehealth platforms to be frustrating. Kelsey, a 39-year old mother, like many others, has minimal experience using virtual platforms; she said,

“*…[I] had to get my daughter*, *who was in college*, *to help walk me through it… definitely frustrating*, *so I’m glad that there was a phone option and not just a zoom meeting*.*”*

Fifth, patients expressed less autonomy in selecting their preferred/usual providers. Participants expressed frustration when they were unable to speak with their usual providers and had to meet with new physicians instead. According to Suzanne, a 41-year old mother,

*My regular doctor knows all my case history*, *so it was really easy to communicate with her; she would understand exactly what I was telling her*, *or even if I had questions from the last visit*, *she would just*, *you know*, *get it very quickly*. *But the same thing*, *when it came to a new provider*, *I had to explain from the very beginning*. *And some of the things she didn’t know about*. *So that was a bit tough and a concern*.

Sixth, missing the therapeutic benefits of the physical presence of physicians. Patients expressed that with telehealth, they lose the physical reassurance of physicians, and the feel of comfort is less than an in-person visit. Sarah, a 33-year old, expressed her disappointment in using the telehealth platform, stating,

*I felt like it was kind of pointless*. *[My physician] literally was just like ‘Okay*, *do you have any questions or concerns*?*’ and I said ‘no*.*’ … it lasted like few minutes*. *In my last in-person visit*, *we talked for a long time*, *and I felt reassured and went home feeling good about my health*.

### Theme four: 4. Telehealth and its potential future uses

Despite some of the ongoing challenges in telehealth services, some participants perceived telehealth positively and expressed a great potential for this service as a practical and useful health-care tool that could be used in the post-pandemic world, particularly for follow-ups, counseling, and monitoring visits that do not require physical exams.

#### Subthemes: 4. 1 Follow-Ups

Interviews indicated a general consensus that telehealth follow-ups are highly effective as long as there is no need for a physical exam. Kimberly, a 30-year old, stated,

*If it’s for a follow-up visit where they don’t actually have to touch me or anything*, *then I would be fine doing telehealth visits*. *I mean*, *you know*, *as far as the driving and stuff*, *because it’s a four-hour total drive there and back*, *so I mean if they don’t have to touch me*, *I’m totally fine with doing the telehealth*.

Participants also expressed how telehealth could expand access to comprehensive medication management. Kristen, a 29-year old, said,

*“I think that medication adjustments and side effects discussion*, *is what we talk about it a lot in follow up visits*. *So*, *I think that is very easy to do via telehealth*.”

Discussing a test or lab results was highlighted as another potential area to be offered via telehealth, even post-pandemic. Referring to this, Deanna, a 40-year old, stated,

*My provider definitely made sure that if I had any questions or concerns to follow up*, *to call her office*, *and*, *stated you know*, *we could have another telehealth chat*. *She did mention that she’d reach out to me after lab work*, *not only for results but also to make sure that I understand it*. *So*, *I thought that was wonderful*.

Similarly, when discussing test results, Marta, a 28-year old, stated,

“*There are things that I don’t need to be in-person for*, *like if we have to discuss a specific test…as long as I don’t need to be examined*, *I think it should be fine to use telehealth*.*”*

#### Subthemes: 4.2 Counseling

Participants demonstrated that counseling is also a potential service that could be expanded post-pandemic. Katrina, a 39-year old, believes that telehealth is a feasible tool for counseling services; she stated,

*I would say telehealth is good for counseling*, *like discussing birth control would be a great reason to have a telehealth visit*. *I mean*, *honestly*, *I would say for most things*, *especially for the OB*, *it would be nice to maybe even start with the telehealth visit because no one really likes having a gyno exam*. *And I think that if you can just talk to the doctor and then they could hear about it and then you could decide if you need to go in*, *in-person*. *I think it’s a really good place to start*.

Similarly, Sarah, a 33-year old, claimed,

*When you don’t have to actually do the tests*, *or you just want some advice*, *I think telehealth is really useful*. *In obstetrics*, *you have to visit frequently*, *so some of the visits scheduled this way were good because every time having to go there in person without even having many problems would just make the commute time longer*. *So*, *weaving these visits in between*, *I thought it was a good idea*. *In late trimesters*, *when the date is near*, *then you definitely have to go to an in-person visit*.

## Discussion

This study explored the experiences and perceptions of OB patients in relation to using telehealth services. During the semi-structured interviews, four themes emerged in relation to the use of telehealth services among OB patients: the overall experience of using modern telehealth platforms, telehealth and its perceived benefits, telehealth and its perceived challenges, and telehealth and its potential future use. The following section presents a discussion of the four themes in more detail.

### Overall experience of using modern telehealth platforms

Several factors, such as ease of use, simplicity, fewer errors/troubleshooting, are critical factors that contribute towards the success of providing care via telehealth platforms [24]. In this study, most participants acknowledged the ease and the simplicity of using telehealth platforms. Participants described their experience using positive terms such as “simple,” “quick,” and “super easy.” As patients perceived the use of telehealth positively, this will lay the foundation for a wider expansion of telehealth services to this population, particularly in the post-pandemic world.

Previous studies demonstrated that there are ongoing challenges that could hinder the success and usability of technological applications such as telehealth, including age, lack of technology infrastructure, and lack of basic training in utilizing technology [25,26]. Findings in this study demonstrated that telehealth tends to be easy to utilize and comprehend for young patients, as they are regularly immersed with modern technology. In contrast, it seems the older patients often lack basic training in utilizing technology, so they may find the telehealth platforms to be frustrating. This is aligned with emerging data regarding the utilization of telehealth. In a recent report, which analyzes data from 1.1 million patients, younger adults, aged 18 to 44, were found more likely to utilize telehealth in comparison with patients of other ages [26]. Therefore, further studies to examine the impact of age, lack of technology infrastructure, and lack of basic training on the success of telehealth services are needed.

### Perceived benefits

Despite the fast advancement in telehealth practices, the use of telehealth in OB practices remains minimal in comparison to other practices in the US [2]. However, during the COVID-19, the adoption of telehealth as a means to provide remote health-care services to address women’s OB concerns has risen to the forefront worldwide and was a top priority for hospitals and OB practices. One theme that emerged from this study is that telehealth is a helpful health-care tool for patients, as it enables them to have remote access to OB subspecialties. Additionally, it enables them to attend their care visits from the comfort of their home, without having to worry about taking time off from work or school, seeking alternative childcare arrangements, coordinating transportation to/from the doctor’s office, or undergoing through check-in processes and long waiting times. This has aligned with findings from previous reports that telehealth has provided patients with more convenient and alternative care options, particularly during the pandemic [27–29].

Our interviews further highlighted that telehealth played a pivotal role in risk reduction during this pandemic for both patients and providers, preventing unnecessary exposure and minimizing physical interactions between them. Thus, the use of telehealth has enabled hospitals and OB practices to still provide health-care services while keeping their patients and providers safe during the COVID-19 outbreak. These basic findings are consistent with recent research reports, showing that telehealth has played a critical role in risk reduction through facilitating social distancing measures [20,21]. Additionally, it minimizes the impact of patient surges on facilities [30] and saves valuable PPE for direct contact encounters while allowing providers to provide care via telehealth to patients in need [5].

### Perceived challenges

In the field of OB, telehealth presents some challenges in its present stages of development and implementation. Accessibility, technology infrastructure, technological difficulties, network problems, absence of providers’ physical presence, inability to perform physical exams, and less patient autonomy regarding selecting preferred providers are amongst the top reported challenges of current telehealth platforms.

In obstetric care, developing a trusting patient-provider relationship is a critical step in supporting patients through every stage of their care. Yet, in telehealth visits, participants expressed their frustration when they were not able to select their preferred or usual care provider. This could be explained due to some of the pandemic changes that were implemented due to a shortage of providers. Consequently, patients’ autonomy in selecting their preferred provider should be considered when offering telehealth services.

In the traditional world of an in-person visit, patients usually receive and feel various types of assurance from their providers, for example, by touching during a physical exam, being valued, listened to, and being allowed to express their feeling or emotions. However, in telehealth visits, participants expressed their concern about missing the therapeutic value of the presence of providers. Therefore, more time during telehealth visits is needed to allow patients to express their needs and providers to listen to their patients, thus developing a better patient-provider relationship.

In OB care, not all patients are appropriate for telehealth services. In this study, participants expressed frustration when some of them were invited for telehealth visits rather than being asked to come for an in-person visit. This finding indicated that a selection criterion developed and used by a clinician is needed. Additionally, finding a balance between what the clinician thinks and what the patient needs is important to consider when selecting a patient for a telehealth visit. Further studies to examine the best approaches to select more appropriate patients for telehealth are needed.

Technology issues such as lack of digital infrastructure (e.g., wireless blood pressure monitors, glucometers, and activity trackers), extended buffering times and network issues, and lack of basic training in utilizing technology were highlighted by participants as a barrier to the telehealth implementation. In a recent scoping review, telehealth technology issues such as lack of high-speed internet connection and technical issues were the most commonly reported [31]. Unfortunately, those factors hinder the success of implementing telehealth services if not carefully considered in the development and implementation phases.

### Telehealth and its potential future uses

Despite the fact that there are still some challenges facing OB practices to expand telehealth and that this care tool may not be ideal for all types of OB patients, it remains a viable option for some women who may prefer a remote visit and fewer in-person visits. An important theme that emerged from this study is telehealth and its potential future uses. The majority of the participants believed that telehealth is a great tool for visits that do not require physical exams, such as counseling and follow-ups (e.g., medication management, discussing tests or lab results, discussing birth control, or reaching for advice). Having a telehealth option available for women who desire to attend fewer in-person visits for counseling or follow-ups align with OB practices’ long-term goals of reducing barriers to OB care, such as lack of transportation, seeking childcare, and long wait times that require missing work or schooling [32].

### Study limitations

There are few limitations to this qualitative study. The effect of non-participation biases is not known. Experiences and perceptions of patients who did not participate in this study might provide additional insights. Additionally, the study was conducted during a pandemic period where telehealth was quickly implemented; thereby, some of the highlighted challenges in this study could be avoided if the implementation was prior to the pandemic. Despite those limitations, this study provides critical insights on the utilization of telehealth for OB patients, based on qualitative patient perspectives and experiences during the COVID-19 pandemic.

## Conclusion

Telehealth is a feasible and safe health-care tool that is available during these unprecedented times. This study provided qualitative evidence based on patients’ perspectives and experiences. Telehealth can be used for OB patients who do not require physical exams or procedures. Telehealth would be an acceptable and effective health-care tool for follow-ups and consultations, thus enabling patients to attend more remote visits when in-person visits are not necessary. However, the highlighted challenges presented in this study are necessary to be addressed in order for telehealth to meet maximum effectiveness and functionality in the future.
